# Financial Position of the London Hospitals

**Published:** 1923-11

**Authors:** 


### THE LONDON HOSPITALS.

### STATISTICS OF INCOME AND EXPENDITURE

IN our August number we dealt with Sir Napier Burnett's fourth Annual Report on the Voluntary Hospitals of Great Britain, excluding London, which is under the supervision of King Edward's Fund. That Fund has now issued the complementary Statistical Report on the Income and Expenditure of the One Hundred and Thirteen London Hospitals for the year 1922. The foundation of the Report consists of the published accounts of these hospitals and of returns made to the Fund, and, taken all round, the figures show a distinctly improved position.

### More Patients and Increased Work.

Between 1913 and 1920 the beds available increased by 1,042, and the average beds occupied by 563, but between 1920 and 1922 there was a decrease in the beds available of 47, and in the average occupied beds of 187, leaving a net increase in 1922 over 1913 of 995 available beds and 376 average occupied beds. The number of new In-Patients increased between 1913 and 1920 by 20,256, but there was a slight decrease in 1921 as compared with 1920 of 1,456, and in 1922 as compared with 1921 of 1,289, showing a net increase in 1922 over 1913 of 17,511. While the new Out-Patients in 1922 show a decrease of 48,000 as compared with 1913, the Out-Patient attendances have increased by 1,225,000. The average attendances of each Out-Patient were, in 1913, 3.5 ; in 1920, 4.3 ; in 1921, 4.2 ; and in 1922, 4.5.

### Hospital Income and Expenditure.

The income of these 113 hospitals (apart from special distributions) increased from £1,470,000 in 1913 to £2,401,000 in 1920, and £2,566,000 in 1921. In 1922 it decreased slightly to £2,398,000. It must be remembered, however, that the special distributions which are omitted from these figures included in 1922 £243,000 from voluntary sources—the first and second instalments of the proceeds of the Combined Appeal. It was part of the scheme of this appeal that for an agreed period the co-operating hospitals should suspend all independent appeals. It was anticipated therefore that, other things being equal, the income from voluntary sources would be below the normal if the proceeds of the Combined Appeal were excluded, and above the normal if they were included. The difficulties of the Hospitals, as compared with conditions before the War, were produced by increase of expenditure and not by decrease of income. It is all the more satisfactory, therefore, that expenditure now shows a substantial decrease. After having risen from £1,192,000 in 1913 to £2,784,000 in 1920, it fell to £2,776,000 in 1921, and to £2,572,000 in 1922.

### Cost of Working in 1922.

From these figures of Hospital work it will be obvious that the greater part of this fall in expenditure is not due to decrease in work, but to reduction in cost. The decrease in the cost of working, which began in 1921, was greatly accelerated in 1922. The most critical year for the Voluntary Hospitals in London was 1920, and the improvements which began in 1921 continued in 1922. Whereas the aggregate deficit in 1920 was £383,000, and in 1921, £210,000, last year it fell to £174,000—less than half the adverse balance of two years before. While the net aggregate deficit of 113 Hospitals for the year 1920 amounted to about 14 per cent. of the expenditure, the deficit for the year 1921 was about 8 per cent. of the expenditure, and for the year 1922 about 7 per cent. of the expenditure. The improvement may be shown in another way by taking the figures of surpluses and deficits separately. In 1921, 48 hospitals had surpluses for the year amounting to £85,000, and 65 had deficits amounting to £295,000 ; while in 1922, 58 hospitals had surpluses for the year amounting to £67,000, and 55 had deficits amounting to £241,000.

### Comparative Cost of Working.

Tables are given, comparing the cost of working for the more controllable items of expenditure. The object of these tables is to assist hospital managers in controlling expenditure, and avoiding as far as possible increases in cost which are not represented by an increase in the quantity or quality of the work done. The method adopted is to compare the expenditure at each Hospital with the expenditure at other Hospitals, and with the average of those Hospitals with which it is most comparable. Increases which are common to all Hospitals can thus be distinguished from those which are peculiar to any particular institution. It is not suggested, however, that the lowest expenditure is intended to be taken as a model. All the comparisons are with the average, and, even so, high expenditure does not necessarily mean excessive or extravagant expenditure ; but all figures above the average indicate branches of expenditure that deserve the most careful investigation by those concerned. The figures of cost per bed in the Report do not profess to do more than show at which hospitals and to what extent the cost of the more controllable items is above or below the average. The question whether a high cost can be justified can only be answered with the aid of more detailed records and statistics kept by the hospitals themselves. In this connection the Hospital Economy Committee of the King's Fund has for some time had under consideration the question of supplementing the use of the present statistics of money cost of working at hospitals, by the use of statistics of quantities consumed. After consultation with the representatives of the larger General Hospitals, and keeping in sight the requirements of other hospitals, the Committee has prepared and issued a memorandum on quantity statistics, which, it is hoped, will assist hospital managers in their efforts to develop the control of expenditure, and to prevent leakage and waste.

[This Report having reached us at the moment of going to press, we have been unable to do more than present a bare summary of a document of the first importance to all hospital managers. We hope to print a considered review of it in our December number.]

